# Task-driven knowledge graph filtering improves prioritizing drugs for repurposing

**DOI:** 10.1186/s12859-022-04608-y

**Published:** 2022-03-04

**Authors:** Florin Ratajczak, Mitchell Joblin, Martin Ringsquandl, Marcel Hildebrandt

**Affiliations:** 1 Helmholtz Zentrum München Deutsches Forschungszentrum für Gesundheit und Umwelt (GmbH), Munich, Germany; 2 Digital Technology and Innovation, Siemens Healthineers, Erlangen, Germany; 3 Corporate Technology, Siemens AG, Munich, Germany

**Keywords:** Knowledge graphs, Knowledge graph embeddings, Drug repurposing, Link prediction

## Abstract

**Background:**

Drug repurposing aims at finding new targets for already developed drugs. It becomes more relevant as the cost of discovering new drugs steadily increases. To find new potential targets for a drug, an abundance of methods and existing biomedical knowledge from different domains can be leveraged. Recently, knowledge graphs have emerged in the biomedical domain that integrate information about genes, drugs, diseases and other biological domains. Knowledge graphs can be used to predict new connections between compounds and diseases, leveraging the interconnected biomedical data around them. While real world use cases such as drug repurposing are only interested in one specific relation type, widely used knowledge graph embedding models simultaneously optimize over all relation types in the graph. This can lead the models to underfit the data that is most relevant for the desired relation type. For example, if we want to learn embeddings to predict links between compounds and diseases but almost the entirety of relations in the graph is incident to other pairs of entity types, then the resulting embeddings are likely not optimised to predict links between compounds and diseases. We propose a method that leverages domain knowledge in the form of metapaths and use them to filter two biomedical knowledge graphs (Hetionet and DRKG) for the purpose of improving performance on the prediction task of drug repurposing while simultaneously increasing computational efficiency.

**Results:**

We find that our method reduces the number of entities by 60% on Hetionet and 26% on DRKG, while leading to an improvement in prediction performance of up to 40.8% on Hetionet and 14.2% on DRKG, with an average improvement of 20.6% on Hetionet and 8.9% on DRKG. Additionally, prioritization of antiviral compounds for SARS CoV-2 improves after task-driven filtering is applied.

**Conclusion:**

Knowledge graphs contain facts that are counter productive for specific tasks, in our case drug repurposing. We also demonstrate that these facts can be removed, resulting in an improved performance in that task and a more efficient learning process.

**Supplementary Information:**

The online version contains supplementary material available at 10.1186/s12859-022-04608-y.

## Background

Drug repurposing (also known as drug repositioning) involves the strategy of finding new indications for already approved drugs outside of their original indications [1]. It has several advantages over the *de novo* drug development, including shortening the timespan of development as well as saving costs [2, 3]. Historically, drug repurposing has lacked systematic approaches. The identification of new indications for a drug was largely due to casual observations during trials or clinical use [2]. Recently, computational approaches have emerged that are driven by an increasing availability of suitable data [2, 4]. These methods usually scale to a large number of different drugs and diseases and have varying degrees of robustness, but they are also very specific to their domain and thus limited in the amount and type of data they can leverage. This limitation has recently been adressed by the development of datasets that span several domains and are modeled in the form of knowledge graphs, specifically for drug repurposing, and computational approaches that can leverage such data types [5–7].

In general, a knowledge graph (KG) is a collection of facts which are encoded as triples, e.g. (*Aspirin*, *treats*, *Headache*) [8]. In this example, *Aspirin* and *Headache* are entities which are connected by the relationship *treats*. Entities can have types that further describe them and group them into categories. In this example, *Aspirin* is of type *Compound*, whereas *Headache* is of type *Disease*. This allows integrating data from various domains, which makes KGs a highly flexible data structure [8]. Entities can also be connected via different relationships, e.g. entities of the type *Compound* can be connected to entities of type *Gene* in various ways, with *upregulates* and *downregulates* describing diametrically opposed concepts [5].

Recently, considerable effort is directed towards developing specialized machine learning models for KGs [9–13]. While the native representation of a large graph is high-dimensional, these methods aim at projecting the graph into a lower-dimensional latent space in a way that best preserves the graph structure. In most cases, this is achieved by learning distributed vector representations for all entities and relationships which embed these concepts into the latent space. Once the feature vectors, or embeddings, are learned, the models can score triples (*head*, *relationship*, *tail*) by using model-specific scoring functions and yield the probability of the given triple being present in the graph or not. Since the triples in the graph represent true facts, this probability can be interpreted as the probability of the scored triple being a true fact. While triples that are present in the graph should score a higher probability than triples that are absent, it is also possible for triples that are absent to score a high probability. This might indicate a previously undiscovered but true triple, given that the model has learned meaningful representations of the concepts embedded in the latent space. Finding these high-probability undiscovered triples is a task known as link prediction, or KG completion. For drug repurposing, this corresponds to high-probability but absent triples of the type (*Compound*, *treats*, *Disease*), which can then be used to prioritize compound-disease pairings for expert revision and clinical trials.

As with most machine-learning approaches, the data that is used to train the model is key to the performance of the trained model. Both quality and quantity of the data have to be sufficient for the model to extract information and generalize to previously unseen examples. Since it is nontrivial to judge the quality of the data in a graph context, most approaches focus on quantity, which leads to increasingly larger KGs. KGs such as WikiData [14], BabelNet [15] or Yago [16] already span millions of entities and billions of facts [8]. As the availability of knowledge grows in the biomedical domain, more databases are compiled into larger KGs [5, 7]. The underlying assumption is that a larger KG with more entities and relations will likely be better for most, if not all, purposes. Knowledge graph embedding (KGE) methods aim at simultaneously optimizing predictions for all relations that are present in a graph. However, in real applications such as drug repurposing, it is usually very few or just one specific relation that is of interest. Other real-world use cases on KGs that focus on one task only are predicting the involvement of compounds in gene regulation [17] and compound side effects [18]. It is therefore possible that while optimizing for all relations, the KGE model underfits the data on the relation of relevance, especially when that relation is underrepresented in the graph. For example, assume we want to predict new triples of type (*Compound*, *treats*, *Disease*), but the vast majority of entities in the graph are of type Gene and the vast majority of relations involve entities of the type Gene. In this case the loss term would mostly reflect how well the model predicts relations incident to genes. This, in turn, would lead to embeddings which are optimised for predicting these majority relations and not for predicting relations between compounds and diseases, which are usually very rare. This implies that by tailoring the KG to a specific task the performance of the model in that task can be improved. We aim at investigating this conjecture by deliberately and programmatically removing entities and triples from two biomedical KGs and analyzing the impact thereof on training and performance in a link prediction task for drug repurposing. We realize this by applying a metapath based filtering approach which allows us to inject domain knowledge about higher order relationships of the prediction task into the filtering procedure and create task-specific KGs.

We make the following contributions:

- We show that two large biomedical KGs contain facts that are not only irrelevant, but even consistently reduce prediction performance for drug repurposing.
- We propose a method to filter a KG based on metapaths that retains facts which are supportive to the prediction task and results in up to 60% reduction in the data set size.
- We conduct an empirical study on 5 KGE approaches and show that our method leads to a consistent improvement in terms of prediction performance (up to 41%).

### Related work

KGE models have gained popularity for drug repurposing lately. Similar embedding methods have been employed by [19], although with a focus on repurposing drugs for Diabetes Mellitus. In [6] the authors of the Hetionet graph database have utilized a custom path-based metric in combination of regularized linear regression for drug repurposing on Hetionet. Drug2Ways [20] leverages paths of causal relations on biomedical KGs for drug discovery while [21] uses neighbourhood information around compounds and diseases for drug repurposing. These methods use paths and neighbourhoods around compounds and diseases in the graph directly as a part of their model’s training. Our approach on the other hand uses metapaths to inject domain knowledge into a task-oriented filtering process. This allows us to use the resulting modified graph to train any machine learning model that operates on graphs. [22] have compiled the Global Network of Biomedical Relationships (GNBR), a biomedical KG that is also incorporated into the Drug Repurposing Knowledge Graph (DRKG) which is used in this work, to predict new treatments for rare diseases using KGEs. KGs have also been used in the adjacent fields of drug repurposing, like predicting interactions between drugs and genes [23], diseases and genes [5, 24] or polypharmacy side effects [25]. There have also been endeavours for large-scale computational drug repurposing specifically for COVID-19 (coronavirus disease) [26, 27], a viral disease caused by the severe acute respiratory syndrome coronavirus-2 (SARS CoV2), some also involving graphs [28–30].

### Preliminaries

KGs consist of an entity set ${\mathcal{E}}$ and a set of binary relations ${\mathcal{R}}$. Each entity $e \in {\mathcal{E}}$ is assigned a type in ${\mathcal{T}}$ according to a mapping $\phi : {\mathcal{E}} \rightarrow {\mathcal{T}}$. While every entity is unique, multiple entities can be assigned the same type. For example, while there is only one entity *Aspirin* in the graph, which is of the type *Compound*, there can be multiple entities of the type *Compound* in the graph. We can define a KG in terms of a set of triples $\mathcal {KG} \subset {\mathcal{E}} \times {\mathcal{R}} \times {\mathcal{E}}$ of the form of (*h*, *r*, *t*). The neighbourhood *N*(*e*) of an entity *e* is the set of entities that are adjacent to *e*. Every triple contained in the graph is considered a true fact and every triple not contained in the graph taken to be unknown (i.e., the open world assumption).

## Results

### Reduction in dataset size

**Fig. 1:**
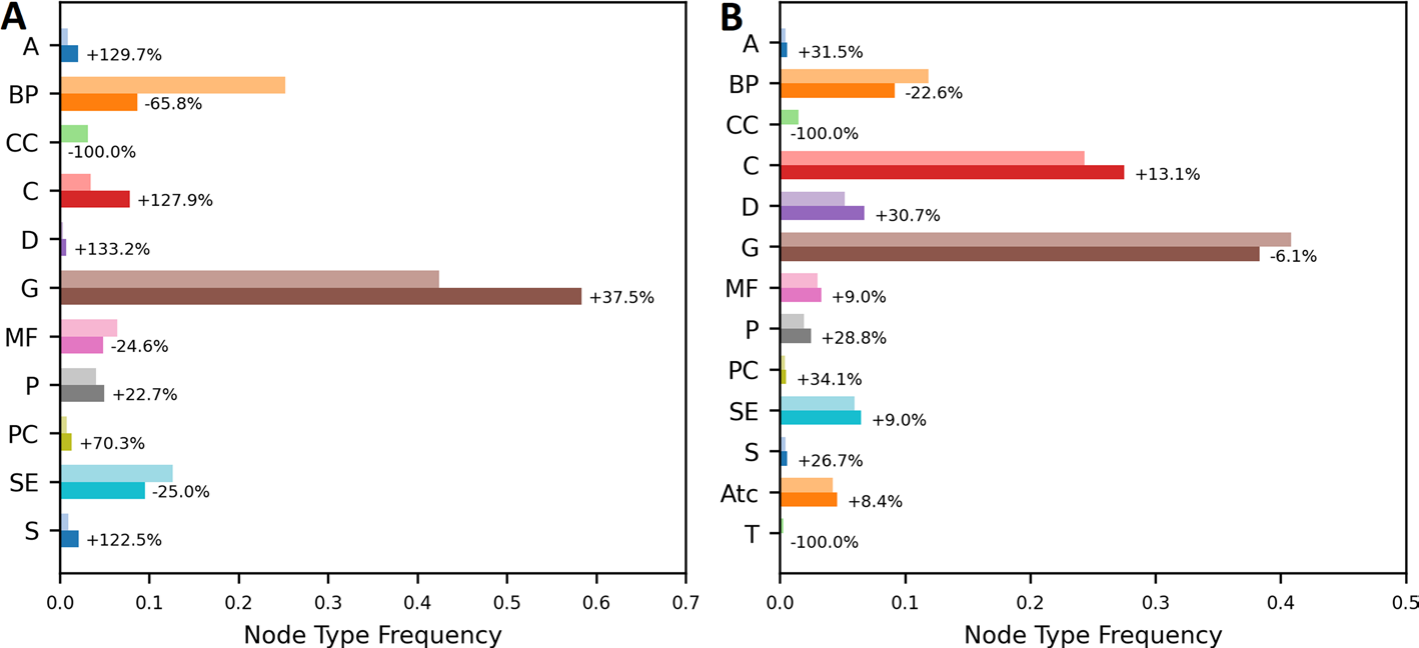
Relative frequencies of node types in **A** Hetionet and **B** DRKG. The lighter bars denote the frequencies in the original datasets wheras the darker bars denote the frequencies in the modified dataset. The change in relative frequency from the original to the modified datasets is denoted as percentages at the end of the bars. A: Anatomy, BP: Biological Process, CC: Cellular Component, C: Compound, D: Disease, G: Gene, MF: Metabolic Function, P: Pathway, PC: Pharmacologic Class, SE: Side Effect, S: Symptom, Atc: Atc, T: Tax

In Hetionet, from the initial 45,158 entities and 2,249,807 triples, only 19,364 and 1,516,799 remain after applying our metapath-based filtering method (see Table 1). When it comes to the entities involved in the prediction task, from the initial 1538 entities of type compound and 136 entities of type disease, only 1503 compound entities and 136 disease entities remain after filtering. In DRKG, from the initial 96,121 entities and 5,874,261 triples only 57,592 and 4,748,143 remain after filtering (see Table 1). From the initial 23,347 compounds and 4,952 diseases, 19,633 and 4,813 remain after filtering. In both datasets, the relative frequency of entities with types Compound and Disease increases after filtering (see Fig. 1). This effect is more pronounced on Hetionet than on DRKG. Other Node types that increase in frequency after the modification in both datasets are Anatomy, Pathway, Pharmacologic Class and Symptom, which all are closely related to the target domain. Node types that are uniformly reduced are Biological Process and Cellular Component. These node types refer to broader biological concepts and are not as domain specific.

**Table 1:** Effects of downsampling on the Number of entities and relations and the average degree

Dataset	Observed variable	Original	Modified	%-Change
Hetionet	Entities (k)	47	19	$-60$
	Triples (M)	2.2	1.5	$-32$
	Avg. degree	24	39	$+65$
DRKG	Entities (k)	96	71	$-26$
	Triples (M)	5.8	5.4	$-7$
	Avg. degree	61	77	$+26$

In summary, applying our method to both KGs results in a significant reduction in both the number of entities and triples. Interestingly, the filtering process seems to increase the relative presence of the two entities types relevant to our prediction task (i.e., compounds and diseases) and of entity types that are closely related to it.

### Impact on training

**Fig. 2:**
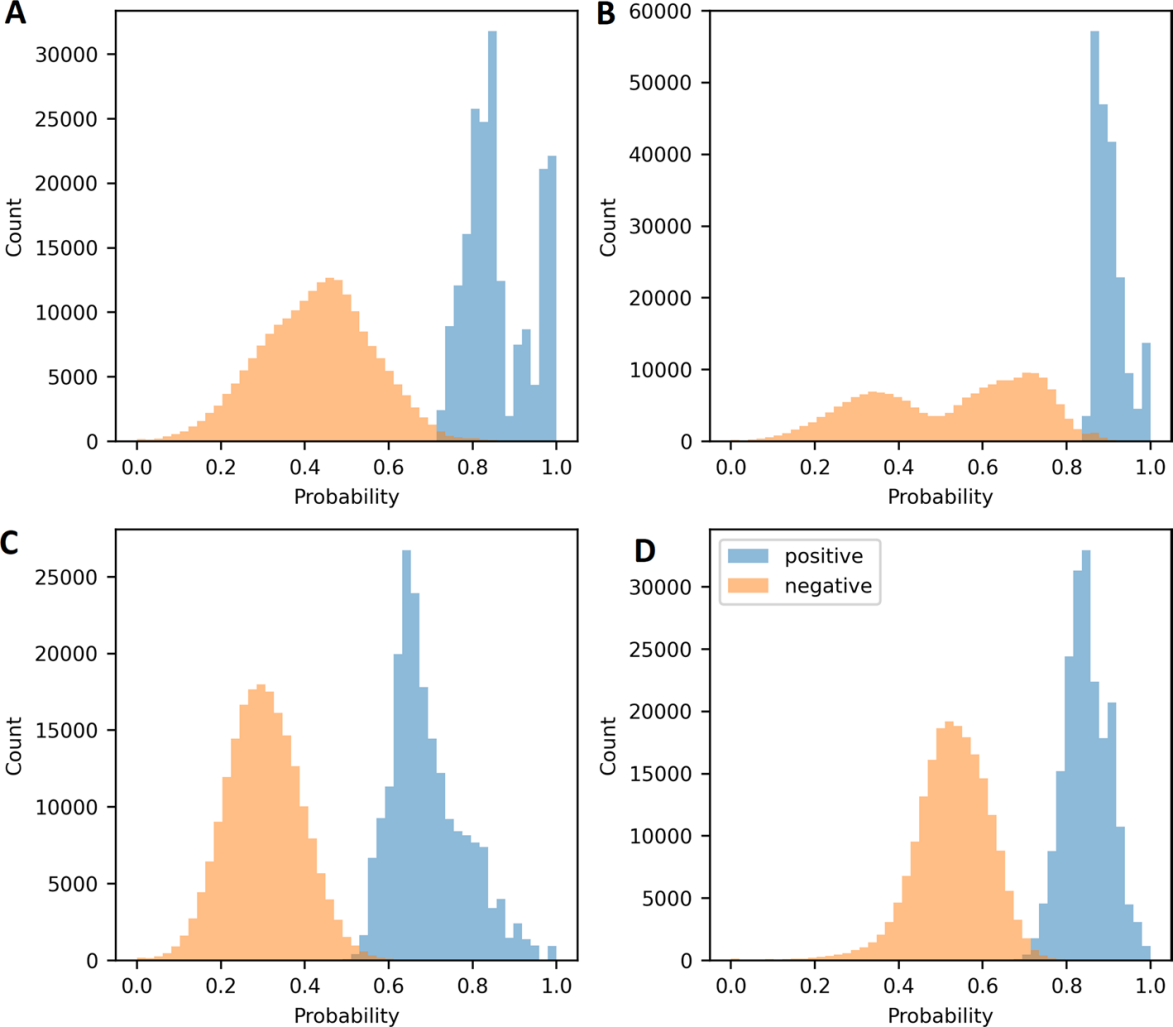
Predicted probabilities of positive and negative relations on the original dataset (**A**, **C**) and the modified dataset (**B**, **D**) of Hetionet (**A**, **B**) and DRKG (**C**, **D**). Shown are 200.000 positive and 200.000 negative relations from the training set, scored and batch normalized in batches of 2000 each. The predictions are obtained from the best performing ComplEx models

Our graph filtering procedure impacts the samples drawn by the negative sampler during training (see Fig. 2), which are used to calculate the loss (see “Post-training analyses” section). Both the negative and the positive samples achieve higher scores on average on the modified dataset. In addition, the entropy as a measure of distance between the two distributions increases from 0.104 on the original dataset to 0.120 on the modified dataset on Hetionet. The increase in entropy taken together with the change to a bimodal distribution indicate that the negative samples drawn after modifying the dataset come from two underlying distributions, one being easier and one being harder to distinguish from the positive samples (see Fig. 2B). In comparison, the negative samples drawn from the original dataset have a uniform distribution and receive lower average probabilites (see Fig. 2A). On DRKG, the entropy between the positive and negative samples decreases from 0.074 on the original dataset to 0.032 on the modified version of DRKG. This indicates that the distributions become more alike, meaning that the negative samples become harder to distinguish from the positive samples (see Fig. 2C, D).

**Fig. 3:**
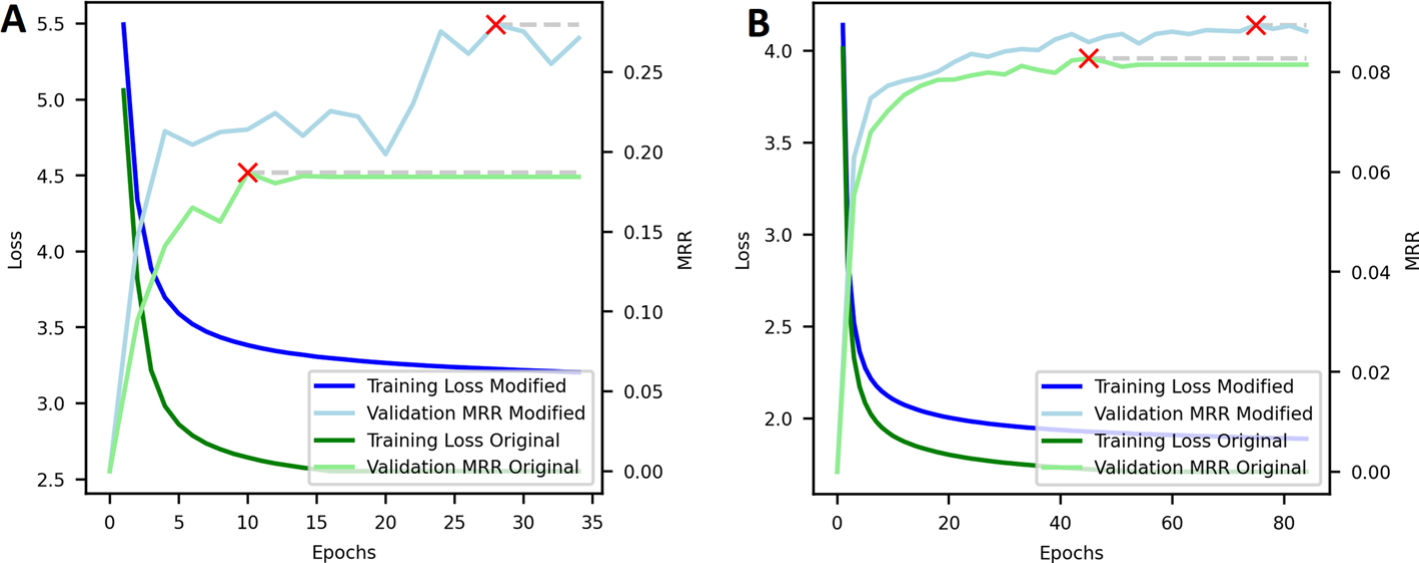
Training loss and evaluation performance curves for the best performing ComplEx models on the original and the modified dataset of Hetionet (**A**) and DRKG (**B**). The red crosses indicate at which epoch the model achieved the best evaluation score before being early stopped three epochs later. The dashed grey lines demonstrate the performance of the best performing model for easier comparison

The training loss is higher on the modified dataset than on the original dataset (see Fig. 3). Conversely, the evaluation performance on the validation set reaches equal levels after fewer epochs and higher global optima on the modified dataset compared to the original dataset. This indicates a higher learning efficiency on the modified dataset. On the original dataset, the evaluation performance begins to saturate earlier, resulting in an earlier stop and worse global optima compared to the modified dataset. The higher training loss indicates that the optimization for the average task in the graph is becoming worse, while the higher evaluation performance indicates that the optimization for drug repurposing improves. This shows that the modification of the dataset is not a free lunch, but instead a trade-off between worse performance on the average tasks but better performance in the task of interest.

### Increased performance

The performances of almost all models have increased after metapath-based filtering (see Table 2, for additional performance metrics see Additional file 3: Tables S6–S7). On Hetionet the performance change ranges from $+6.4\%$ up to $+40.8\%$, wheras on DRKG it ranges from $+3.6\%$ to an increase of +14.2%. Like with the downsampling, the effects of our method are less pronounced on DRKG than on Hetionet.

**Table 2:** Effects of downsampling on the performance

Dataset	Model	Original (MRR)	Modified (MRR)	%-Change
Hetionet	TransE	0.2232	0.2374	$+6.4$
	DistMult	0.2280	0.2517	$+9.3$
	ComplEx	0.1975	0.2782	$+40.8$
	RESCAL	0.2428	0.2940	$+21.1$
	ConvE	0.1312	0.1647	$+25.5$
	Random	0.0343	0.0331	$-3.5$
DRKG	TransE	0.0822	0.0938	$+14.2$
	DistMult	0.0899	0.0931	$+3.6$
	ComplEx	0.0896	0.0950	$+5.9$
	RESCAL	0.0578*	0.0650*	$+12.4$
	ConvE	0.0618*	0.0649*	$+4.9$
	Random	0.0102	0.0086	$-16.7$

*) Due to computational constraints, no hyperparameter optimization (HPO) has been performed for these models. The hyperparameter setting was chosen based on the results of the HPO on Hetionet

### SARS-CoV2 case study

**Fig. 4:**
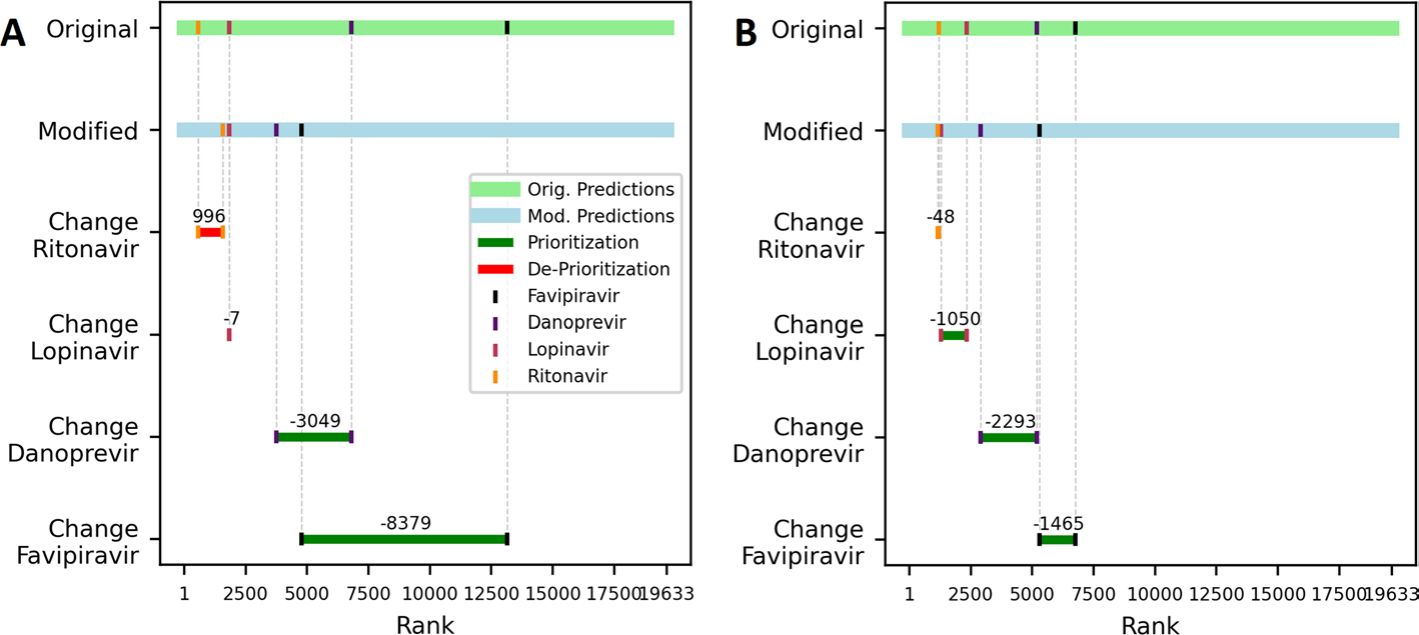
Predicted ranks of four antiviral Compounds and SARS-CoV2 entities compared to the ranks of all Compounds. The light green and blue bars show the ranks of predictions of the type (*SARS CoV2 entity*, *treats*$^{-1}$, *Compound*) for all Compounds in the graph, using the original and the modified dataset for training. The predicted ranks of the four antiviral Compounds are highlighted. The red and dark green bars show the change in the predicted rank of a given antiviral compound when the model was trained on the original dataset versus when the model was trained on the modified dataset. In **A**, the SARS-CoV2 entity is the SARS-CoV2 Spike entity. **B** shows the mean ranks of all 27 SARS-CoV-entities in the graph with each of the four antiviral compounds (for all individual ranks see Additional file 1: Table S3). Expected median rank is 9816, lower ranks indicate higher-than-average probabilities of treatment

As shown in Fig. 4A, training the original dataset does not allow the model to adequately capture the relevance of favipiravir and danoprevir in its role as an antiviral compound against SARS-CoV2. The modified dataset however allows the model to prioritize all antiviral compounds over the median rank. It also prioritizes the antiviral compounds stronger than the model trained on the original dataset, especially visible in danoprevir and favipravir. Only ritonavir is less prioritized by the model trained on the modified dataset than by the model tained on the original dataset. Taking into account the average rank of the four compounds with all 27 SARS-CoV entites in the graph, it is clear that the modified dataset helps the model to prioritize the antiviral compounds (see Fig. 4B). All compounds receive lower mean ranks when the model is trained on the modified dataset than when it is trained on the original dataset.

### Cancer case study

As shown in Table 3, the modified dataset is better suited than the original dataset at predicting treatments that have not been included in the training set for three common types of cancer and cancer in general. This further emphasizes the increase in performance in the drug repurposing task obtained by modifying the dataset. For additional performance metrics see Additional file 3: Table S15.

**Table 3:** Effects of downsampling on the performance of predicting treatments for cancer

Disease	n	Original (MRR)	Modified (MRR)	%-Change
Cancer (general)	141	0.0154	0.0189	$+22.7$
Breast cancer	65	0.0137	0.0245	$+78.8$
Lung cancer	17	0.0177	0.0355	$+100.5$
Colon cancer	16	0.0144	0.0160	$+11.1$

## Discussion

Our experiments show that our method increases the average degree per entity (see Table 1), thus increasing the neighbourhood richness, which produces entity embeddings that achieve higher performance on the drug repurposing task. It also increases the relative proportion of *Compound* and *Disease* entities, thus making them have a greater impact on the overall loss. On DRKG, it also removes a significant percentage of Compounds, indicating that these Compounds were not densely connected to any disease by any of the given metapaths. However, since these entities are not considered during the evaluation of the performance, their removal is not responsible for the improvements reported in Table 2. If such a model would be used in production, however, it would be of utmost importance to remove such possibly biased and low-confidence predictions, as is done by our task-driven metapath filtering procedure.

The increase in performance reported in Table 2 is possibly due to the negative samples that are generated during training becoming more similar to the positive samples. In the initial graphs, the unconstrained topology makes it possible that certain entities are in great distance or even unreachable from a *Compound* or *Disease* entity. This makes it possible for the negative sampling to draw negative samples that are easily distinguishable from positive samples and that carry no information for our prediction task. On the modified graphs however, the walking strategy applied during filtering makes sure that all entities are at most half of the length of the longest metapath away from a *Compound* or *Disease* entity. This makes the samples generated by the negative sampler more alike to the positive samples that they are being compared to and increases their informative value relative to our prediction task, thus increasing learning efficiency. In Fig. 2 it can be seen that after the filtering, the negative samples move closer to the predictions of the positive samples. The predicted probabilities of the positive samples on the other hand move closer to 1, resulting in more confident predictions. This scenario makes the learning problem harder, which results in a higher training loss (see Fig. 3). The more confident predictions of positive relations alongside with the fact that in the modified dataset most of the graph is in direct proximity of entities of the type *Compound* and *Disease* simultaneously results in a higher evaluation performance on the validation and test holdout sets, as can be seen in Fig. 3 and Table 2, respectively.

Entities that are very densely connected to Compounds and Diseases and are intuitively related to our prediction task, like Pharmacological Class, Side Effects and Symptoms, are not heavily downsampled and increase in relative frequency (see Fig. 1). Cellular Components and Biological Processes on the other hand, where a significant proportion of the entities are not expected to play a vital role in drug-disease mechanisms, are uniformly reduced in relative frequency. Genes are heavily reduced as well by almost 50% (see Additional file 1: Tables S1 and S2) but retain their relative frequency in the graph (see Fig. 1). This indicates that, while Genes play a crucial role in the mechanistic connections between Compounds and Diseases, a preliminary selection of certain Genes might be helpful for future studies of biological networks. This is further emphasized by the results of our ablation experiments (see Additional file 3: Tables S9–S12), which show that the performance is usually reduced when all entities of a certain type are removed from the graph. However, when all genes are removed, the performance increases. This is particularly interesting since most KGs used for drug repurposing use genes as a central hub which connects various types of other entities [6, 7, 19–21, 31]. It is worth noting here that path-based methods which are specialized for drug-repurposing such as [6, 19–21] are likely to suffer from entirely removing this central entity type, since the majority of paths from compounds to diseases cross through one or multiple genes. However, as we have shown, popular KGE models are dependent on the negative sampling process to find informative examples in order to guide the learning. Since not all genes are expected to play a role in drug-disease interactions, this entity type is expected to have a lower signal-to-noise ratio than other entity types, e.g. symptoms, while simultaneously being very frequent. These two points together might tip the scale towards genes being unhelpful during training with negative sampling regimes and might explain why path-based methods often outperform KGE models for drug-repurposing on a gene-centric KG [19]. Additionally, especially in DRKG, removing all genes disconnects a large portion of compounds and diseases (see Additional file 3: Table S8), thus making them easy-to-predict negative samples. This is expected to artificially increase performance on the positive samples but simultaneously denies potential insights gained from those disconnected compounds and diseases. With the integration of Hetionet and DRKG into general KGE frameworks [32] and regular KGE models gaining more traction in drug repurposing [19, 31], the influence of genes on the drug repurposing performance of these models remains to be thoroughly investigated. However, the genetic underpinnings of drug-disease interactions provide crucial interpretational guidance on the results of drug repurposing studies. Thus, simply removing all genes from these KGs has a high cost attached to it and is likely not favourable, even though it improves prediction performance. This, again, highlights the demand for more nuanced, adaptable and task-oriented KGE models.

Since the walker has a higher chance to pass through a densely connected entity compared to a sparsely connected entity, well established entities are favoured during the task-driven metapath filtering. This relies on the assumption that e.g. a gene that has been well researched will have more adjacent relations than a gene has not been researched that well yet, since most of its possible connections to compounds and diseases, or its roles in metabolic functions, are not discovered yet. Applying this example also to other entity types, the stochastic nature of our task-driven metapath filtering method might provide a rudimentary form of retaining established entities while filtering out less established entities.

Since lopinavir and ritonavir are well established antiviral drugs, it is not surprising that they earn the lowest ranks from the four selected compounds. However, the results shown in Fig. 4 indicate that the model trained on the original dataset does not adequately capture the relevance of favipiravir and danoprevir in its role as antiviral compounds against SARS-CoV2. The modified dataset however allows the model to effectively prioritize these compounds. Lopinavir and ritonavir appear not to be effective against SARS-CoV2, despite being an established treatment for other viral diseases [33–35]. Favipiravir and danoprevir on the other hand show promise as treatment options for SARS-CoV2 [34, 36, 37], which further emphasizes the relevance of the increased prioritization of these compounds by modifying the dataset. Since none of the four antiviral compounds is directly connected to any of the 27 SARS-CoV2 entities in the graph, this illustrates that the modified dataset is better suited to generate new insights and prioritize drugs for repurposing than the original dataset, even when the disease does not yet have any treatment present in the graph at all.

However, as indicated by comparing the results of the two datasets, for the degree of downsampling as well as for the performance, the improvements generated by this downsampling method are greater in Hetionet than they are for DRKG. Compared to Hetionet, the relative reduction of entities in the graph as well as the relative increase in the average entity degree is smaller in DRKG (see Table 1). This is possibly due to the fact that entities of the types *Compound* and *Disease* as well as *treats*-relations are severely underrepresented in Hetionet while they represent a large part of the graph in DRKG (see Fig. 1). This indicates that the effect of the metapath-based filtering is more pronounced the more the relevant entities and relations are underrepresented in the graph. Over time KGs will expand into an increasing number of domains and contain diversified sets of entity types and relations. This will lead to individual relations and entity types being increasingly underrepresented in comparison to the whole graphs. Since most real world use cases like drug repurposing focus on a small set of relations, underrepresentation of the relevant relations will be a widespread problem. Especially since in most KG scenarios scarce data of interest is enriched with large quantities of supporting data, the portion of the data that is most valuable for the target domain is almost always heavily underrepresented. Therefore, methods to alleviate this underrepresentation problem and to identify and remove irrelevant information on a per-task basis will be crucial. Finally, the modification of future KGs will be necessary not only from a theoretical and performance point of view, but also regarding practical aspects like training time and memory requirements. If the information gathered in KGs expands to an increasing amount of domains, better methods to carve out task-specific subgraphs from these emerging supergraphs will become indispensable.

## Conclusion

We present a metapath-based approach to KG filtering which allows one to include domain knowledge in the form of metapaths. The selection of metapaths can be adapted to modify a KG and tailor it for a specific task, which we demonstrate by applying it for the task of drug repurposing. We present evidence that a proportion of up to 60% of a KG can be removed by this filtering method while simultaneously improving the performance in the task by up to 40%. This result demonstrates that the removed information is in fact counterproductive to learning embeddings for the specific task. During training, the loss on the modified graph is higher, while simultaneously the link prediction performance for drug repurposing is improved. This indicates that, while the model has worse performance on the average task, it has an improved performance in the relevant task, i.e. drug repurposing. This is in part attributed to the entity type of relevance becoming more frequent, an increase in the average degree and the samples drawn during training becoming more informative to the task. Furthermore it is shown that the models trained on the modified graph reach equal performance earlier than the models trained on the original graphs, which emphasizes a higher learning efficiency, while simultaneously consuming less resources. Given the improved prediction performance in drug repurposing in general and in the specific case of antiviral compounds and SARS-CoV2 it can be concluded that the task-specific modified graph is less general and more specific to the use case than the original graph.

Future work can be applied in several directions. While we show that KGs contain counterproductive information that can be reduced to improve performance in a certain task, this has to be done as a preliminary step before training the model. Allowing a model to prune a graph by removing entities or gradually reducing their impact during training, possibly guided by domain knowledge, would be the next logical step. A similar approach has recently been developed for graph neural networks [38]. A different direction of future work is to further investigate the trade-off between different intensities of filtering and changes in performance. This would be necessary to formulate the filtering as an optimization problem and to possibly find global optima for a given task and scenario.

## Methods

### Task-driven metapath filtering

We propose a method to filter a KG based on a set of metapaths *M*. The metapaths $m_1,m_2,\ldots ,m_l \in M$ can be chosen to incorporate domain knowledge in the filtering process. They further enable us to constrain the maximum distance between every node in the graph and an entity of the types of interest. In the example of drug repurposing, the entity types of interest are Compounds and Diseases. Thus, we can choose the metapaths so that all the entities in the graph are within a certain distance of Compounds and Disease and are necessarily connected to them via entities of the types of our choice.

Formally, a metapath *m* is a defined sequence of entity types $t \in T$. Given a metapath $m=(t_1, t_2,\ldots , t_{n-1}, t_n)$ of length *n*, a random metapath walker can start at any entity $e_1$ of the type $t_1$. In the next step, the walker samples all entities of the type $t_2$ in the neighbourhood $N(e_1)$ of $e_1$ and selects one at random. Now the walker samples all entities of the type $t_3$ in the neighbourhood $N(e_2)$ of $e_2$ and selects one at random again. This procedure is repeated until the walker either reaches an entity $e_n$ of type $t_n$ or arrives at an entity $e_i$ that has no entity $e_{i+1}$ of the required type $t_{i+1}$ in his neighbourhood $N(e_i)$. In both cases, the walker stops and starts a new, unrelated walk at an entity of type $t_1$. After the walker started a predefined number of starts at each entity of type $t_1$, the procedure is repeated and the metapath is traversed in the opposite direction, starting at entity type $t_n$.

This procedure yields a set of complete walks ${\mathcal{W}}_{\textit{complete}}$, where the walker reached an entity $e_n$ of type $t_n$, and a set of prematurely stopped walks ${\mathcal{W}}_{\textit{stopped}}$, where the walker stopped at an entity $e_i$ that has no required next node in its neighbourhood. To avoid small disconnected graphs, two walks in ${\mathcal{W}}_{\textit{complete}}$ are concatenated if the last entity in one walk is also the first entity in another walk, reminiscent to the way long tracks are built up in the popular game dominoes. This concatenation procedure is continued until the concatenated walk reaches a predefined length. Then, the concatenation stops and the concatenated walk is placed in ${\mathcal{W}}_{\textit{concatenated}}$. This procedure is repeated until ${\mathcal{W}}_{\textit{concatenated}}$ contains a predefined number of concatenated walks. Now, a subset of entities ${\mathcal{E}}^{'} = \left\{ e | e \in W\,\hbox {for some walk}\,W \in {\mathcal{W}}_{\textit{concatenated}} \right\}$ is taken, clearly ${\mathcal{E}}' \subset {\mathcal{E}}$. In other words, only entities *e* that appear at least once in the concatenated walks ${\mathcal{W}}_{\textit{concatenated}}$ are assigned to ${\mathcal{E}}'$. This subset ${\mathcal{E}}'$ is further called the modified version of the original set of entities ${\mathcal{E}}$. Thus, a new task specific graph ${\mathcal{KG}}' \subset {\mathcal{E}}' \times {\mathcal{R}}' \times {\mathcal{E}}'$ is created which is a modification of the original graph ${\mathcal{KG}} \subset {\mathcal{E}} \times {\mathcal{R}} \times {\mathcal{E}}$.

### Experimental setup

To asses the effectiveness of our method, we have produced modified versions of two datasets by applying walk-based filtering as described in “Task-driven metapath filtering” section. The effect on both of the datasets has been evaluated by training five different KGE models on the original datasets and on the task specific modified datasets.

#### Datasets

**Fig. 5:**
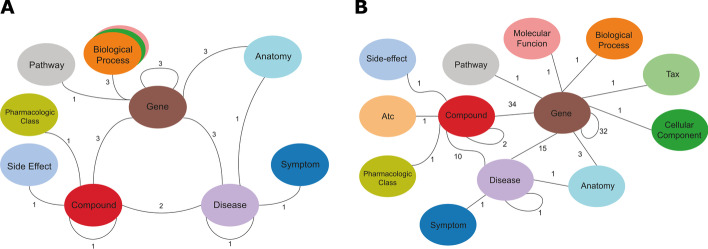
**A** Hetionet. **B** DRKG. The graphs are displayed as metagraphs, where each entity of a type is collapsed to one meta-entity which are connected if there exists at least one relation type between them. The numbers at the relations show how many different types of relations between the given entity types exist. Figures originally published in [6, 7], modified

Hetionet is a biological KG constructed from the integration of 29 publicly available databases [5, 6]. From these databases, a heterogeneous graph has been constructed following the schema displayed in Fig. 5 A. In total it consists of 47,031 entities of 11 different types (see Table 4), which are connected by 2,250,197 relations of 24 types (see Fig. 4). DRKG [7] is a superset of several KGs from the biomedical domain and is the largest compilation of biomedical knowledge in graph form to date. It integrates Hetionet, GNBR, DrugBank, String, IntAct, DGIdb and individual publications into one comprehensive biological KG with 97,238 entities of 13 types (see Table 4) and 5,874,261 relations of 107 types (see Fig. 5B).

**Table 4:** Hetionet and DRKG Entity Types. All figures are before any preprocessing has taken place. [6, 7]

Entity type	Hetionet	DRKG
Anatomy	402	400
Atc	0	4048
Biological process	11,381	11,381
Cellular component	1391	1391
Compound	1552	24,313
Disease	137	5103
Gene	20,945	39,220
Molecular function	2884	2884
Pathway	1822	1822
Pharmacologic class	345	345
Side effect	5734	5701
Symptom	438	415
Tax	0	215
Total	47,031	97,238

#### Dataset preprocessing

*Removal of ambiguous relations* To reduce ambiguity in the results, all relations between the entities that are used during evaluation should be of the desired type. In our case, the entity types are *Compound* and *Disease*, and the desired relation between these entity types is *treats*. All relations between entities of the type *Compound* and *Disease* that are not *treats* are removed. In Hetionet, this only removes the *palliates* relation. In DRKG, the removed relations are *Pa* (alleviates, reduces), *Sa* (side effect/adverse event), *J* (role in disease pathogenesis), *Mp* (biomarkers of disease progression) from GNBR in addition to the *palliates* relations from Hetionet. The remaining relations between compounds and diseases are *T* (treatment/therapy), *C* (inhibits cell growth), *Pr* (prevents, suppresses) from GNBR, *treats* from Drugbank and *treats* from Hetionet. Although *C* and *Pr* from GNBR are not worded as treaments, their character is more curative than palliative and are therefore included into the analysis. During this step, 21,447 triples are removed from DRKG and 390 triples are removed from Hetionet. We have furthermore evaluated Hetionet and the relations involved in DRKG that have been imported from Hetionet for contradictory triples. We have found no contradictory triples for the three variants of *upregulates*/*downregulates*, meaning for two given entities there exist no triples like $({\textit{Entity}}_1,{\textit{upregulates}}, {\textit{Entity}}_2)$ and $({\textit{Entity}}_1, {\textit{downregulates}}, {\textit{Entity}}_2)$ simultaneously.

*Unification of related relations for prediction* In the case of DRKG, there are several relations between entities of the type *Compound* and *Disease* that are described as *treats* or similar concepts. To allow the information learned from one of these relations to be transferred to the others, all remaining non-ambiguous relations between entities of the type *Compound* and *Disease* are replaced by a unifying *treats* relation. Since there is an overlap of the various *treats*-like relations, unifying them removes another 1793 triples from the graph. After preprocessing, DRKG contains 96,121 entities, 4952 of which are diseases and 23,347 are compounds.

*Introduction of inverse relations* Since most KGE models represent relations in a directed fashion, inverse relations have been introduced into the graphs. This means that for every relation *r* there is an inverse relation $r^{-1}$ that is connected to the same entities, but in the opposite direction. For example, in addition to the triple (*Compound*, *treats*, *Disease*), there now also exist triples in the opposite direction (*Disease*, *treats*$^{-1}$, *Compound*). To avoid easy-to-infer inverse triples, training, validation and test sets have been constructed first so that every triple can only be in the training, validation or the test set. Only then, inverse relations have been introduced for each set separately.

#### Metapath selection

The metapaths selected for use in our method are based on those used by [6], but omitting the relations and only considering sequences of entity types (see Additional file 2: Table S4). For DRKG, three additional metapaths have been added in the direct neighbourhood of the *Compound* entity type to also include the additional entity type *Atc* (see Additional file 2: Table S5). The metapaths have been traversed in both directions, once starting from the compounds and once from the diseases. The number of starts for every compound and every disease has been set to 1000 per metapath, the length of the concatenated walks is set to 100 entities and the number of concatenated walks gathered per metapath is set to 5000.

#### KGE methods

We trained five different KGE Models: TransE [9], RESCAL [10], DistMult [11], ComplEx [13] and ConvE [12]. Translational methods, such as the seminal work TransE [9], embed both entities and relations into the same vector space. Subsequently, translational methods consider additive functions to model the actions of relations on entities. Factorization-based approaches such as RESCAL [10], DistMult [11], or ComplEx [13] define multiplicative scoring functions on the entity embeddings. Specifically, each relations $r \in {\mathcal{R}}$ induces a parametrized bi-linear form $(\cdot , \cdot )_r:\mathbb{R}^d\times \mathbb{R}^d \rightarrow \mathbb{R}$. Next to methods that combine graph neural networks and KGE methods, the method ConvE [12] is commonly employed for knowledge base completion (KBC). ConvE [12] learns 2D-convolutional networks over the embedding space to obtain expressive feature maps that serve as input to a bilinear product that scores candidate triples.

#### Performance measure

The link prediction performance is evaluated only on triples of the type (*Compound*, *treats*, *Disease*) and (*Disease*, *treats*$^{-1}$, *Compound*). Negative samples are generated by permuting the tail entities and restricting the permuted tail entities to be of the same type as the original tail entity (i.e. for (*Compound*, *treats*, *Disease*), we permute the tail entity only with other Diseases). The metric chosen for evaluation is the filtered Mean Reciprocal Rank (MRR):

1 $$\begin{aligned} {\textit{MRR}} = \frac{1}{|Q|} \sum _{i=1}^{Q}\frac{1}{{\textit{rank}}_i} \end{aligned}$$

where *Q* is the number of queries and ${\textit{rank}}_i$ is the rank of the *i*th correct result according to the scoring function $(h,t)_r$. In our scenario, we have one query for every true triple of the types (*Compound*, *treats*, *Disease*) and (*Disease*, *treats*$^{-1}$, *Compound*). We compare this true triple to false triples with the same head entity and relationship, but permuted tail entities. The permuted tail entities are all entities in the graph with the same type as the true tail entity which are not connected to the head entity by the given relation. Then, predicted probabilities are sorted in descending order and the reciprocal of the rank of the one true triple. The mean of the reciprocals is the MRR. An MRR of 1 means that for every query, the correct result is always on rank 1. It is called filtered because each true triple is compared individually against all the false triples (i.e. observed triples are excluded from the ranking). Thus, multiple true triples for a single head entity don’t influence each other’s ranks. Additional performance measures are introduced in Additional file 3.

Additionally, we only consider entities of the types *Compound* and *Disease* for evaluation that occur in both the full and the filtered graph, regarding both the true and the permuted triples. To evaluate the difficulty of the prediction task we include the performance of a random classifier. To obtain the random classifier results, the scores of a hyperparameter-optimized model for all compound-disease pairs are taken. Then, the ground truth adjacency matrix between Compounds and Diseases is randomized and the performance of the scores on the randomized ground truth is evaluated. This procedure is repeated 100 times and the mean of the performances is taken.

#### Validation

Prior to our metapath-based filtering, two holdout sets have been taken for both of the datasets. The first holdout set is used for the selection of optimal hyperparameters (validation), wheras the second holdout set is used to asses the final performance per model, after all parameters and hyperparameters are fixed (test). On Hetionet, these holdout sets each contain 12.5% of the *treats* relations and their inverses each, whereas on DRKG they each contain 10% of the *treats* relations and their inverses. The remainder of the treats-relations and all other triples in the graph are used for training (see Additional file 3: Table S16). If an entity is removed by the metapath-based filtering procedure, then all triples containing this entity are removed from all holdout sets, resulting in identical holdout sets for the original and the modified datasets.

#### Post-training analyses

To better understand the impact that the filtering procedure has on the training and on the final embeddings, comparable models are chosen after training is finished for further inspection. The models have to produce good results on both Hetionet and DRKG in both the original and modified setting to be chosen. Additionally, the hyperparameter optimization (HPO) has to be completed and the best performing model after HPO is chosen for the post-training analyses.

*Effect on Negative Sampling* During training, negative samples are drawn from the graph by corrupting existing triples, i.e. positive samples. Although the truth value of these negative samples is unknown, they are being treated as false during training (local closed world assumption). The training loss is determined based on comparing the predicted probabilities of the positive (existing) samples to the sampled negative (not existing) samples. Thus, the samples drawn during negative sampling can have a non-negligible effect on the training. The effect of our metapath-based filtering procedure on the samples drawn during negative sampling is examined by drawing 200,000 positive and 200,000 negative samples in batches of 2000 each. The batches are scored using a trained model. The predictions are scaled to be in the interval [0, 1] where 0 indicates a low probability of the triple being true and 1 indicates a high probability of the triple being true. The resulting distributions are compared visually and by entropy.

*SARS CoV2 Case Study* COVID-19 is a current global health challenge and the search for optimal treatment options is still ongoing. To better understand how the change in model performance after filtering can translate into insights, the final models will be compared in how they rank four compounds that have been in the discussion as potential treatments of the disease and all 27 SARS CoV2-related disease entities in DRKG. The chosen compounds are danoprevir, favipiravir, lopinavir and ritonavir, which are all currently investigated as antiviral drugs in the treatment of COVID-19 [37, 39, 40]. The antiviral compound remdesivir is not included in the analysis because it is not present in DRKG. Since the mechanistic target of the antiviral compounds, the RNA-dependent RNA polymerase (RdRp) [41], is not present in DRKG, we focus on the SARS-CoV2 Spike protein entity’s rank and the mean rank over all 27 SARS-CoV2 entities. None of the SARS-CoV2 entities have an adjacent *treats* relation, thus the dataset reflects that they have no known treatment. Each SARS-CoV2 entity is ranked against each of the 19633 remaining compound entities in triples of the form (*SARS CoV2 entity*, *treats*$^{-1}$, *Compound*). The rank of the antiviral compounds compared to all the other compounds in the graph is noted. A low rank reflects a high priority, i.e. a high probability of a *treats*$^{-1}$ relation between the disease and compound entities.

*Cancer Case Study* To further investigate the effect of the metapath-based filtering on drug repurposing for certain diseases, we have analysed how well the models trained either on the original or the modified dataset can predict treatments for different forms of cancer from a holdout set. Therefore, we have selected the entities of four common cancers in DRKG by their MESH ID: Cancer (general) (MESH: D009369), Breast Cancer (MESH: D001943), Lung Cancer (MESH: D008175) and Colon Cancer (MESH: D003110). The four diseases have 141, 65, 17 and 16 treatments in the holdout set, respectively. Each cancer entity is scored against each of the 19633 remaining compound entities in triples of the form (*Cancer entity*, *treats*$^{-1}$, *Compound*).

## Supplementary information

**Additional file 1: Table S1**. Effects of Downsampling per Entity Type in Hetionet. **Table S2**: Effects of Downsampling per Entity Type in DRKG. **Table S3**: Effects of Downsampling on the ranking of antiviral compounds against SARS CoV2-entities. Shown is the rank ofa given SARS-CoV2 entity with a given antiviral compound when the model is trained on the full dataset versus when it is trained on the filtered dataset. The SARS-COV2 entities are ranked against allr emaining 19,633 compound entities by connecting them with a *treats* relation. Rank 1 means the highest probability that the two entities are connected by a *treats* relation, rank 19,633 mean sthe lowest probability, rank 9816 corresponds to the median probability.

**Additional file 2: Table S4**. Metapath Selection. Metapaths that have been selected for random metapath walking. Denoted are the entity types traversed. C: Compound, D: Disease, G: Gene, A: Anatomy, PC: Pharmacologic Class, S: Symptom, BP: Biological Process, MF: Metabolic Function, PW: Pathway, SE: Side Effect. **Table S5**: Additional Metapaths for DRKG. Metapaths selected for DRKG in addition to the metapaths denoted in Table S4. C: Compound, Atc: Atc, D: Disease, G: Gene.

**Additional file 3: Table S6**. Additional performance metrics for Hetionet. **Table S7**: Additional performance metrics for DRKG. **Table S8**: Compound and Disease entities that are disconnected by an Ablation Experiment. **Table S9**: Entity Type Ablation Experiments—Hetionet—TransE. **Table S10**: Entity Type Ablation Experiments—Hetionet—ComplEx. **Table S11**: Entity Type Ablation Experiments—DRKG—TransE. **Table S12**: Entity Type Ablation Experiments—DRKG—ComplEx. **Table S13**: Effects of train/valid/test split on model performance—TransE. **Table S14**: Effects of train/valid/test split on model performance—ComplEx. **Table S15**: Additional performance metrics for the Cancer Case Study. **Table S16**: Sizes of training, validation and test splits.

## Data Availability

The original data from which derivatives are used in this work is available for download at the original author’s terms. For Hetionet, see https://github.com/hetio/hetionet For DRKG, see https://github.com/gnn4dr/DRKG The datasets supporting the conclusions of this article are available in the Zenodo repository, 10.5281/zenodo.5638999 . The implementation of the metapath-based filtering approach is available at https://github.com/fratajcz/metafilter-apply. The walker implementation is based on the StellarGraph framework [42]. The code that is necessary to run the same experiments is available at https://github.com/fratajcz/metafilter-experiment and the datasets and versions thereof are available . The implementation of our experiments is based on the KGE framework LibKGE [43], which we have adapted to our needs.
