# Supplementary material for: Task-driven knowledge graph filtering improves prioritizing drugs for repurposing
Source: BMC Bioinformatics. 2022 Mar 4;23:84. doi: 10.1186/s12859-022-04608-y (PMC8894843; doi:10.1186/s12859-022-04608-y)
Supplement: Supplementary file 1 — Additional file 1: Table S1. Effects of Downsampling per Entity Type in Hetionet. Table S2: Effects of Downsampling per Entity Type in DRKG. Table S3: Effects of Downsampling on the ranking of antiviral compounds against SARS CoV2-entities. Shown is the rank ofa given SARS-CoV2 entity with a given antiviral compound when the model is trained on the full dataset versus when it is trained on the filtered dataset. The SARS-COV2 entities are ranked against allr emaining 19,633 compound entities by connecting them with a treats relation. Rank 1 means the highest probability that the two entities are connected by a treats relation, rank 19,633 mean sthe lowest probability, rank 9816 corresponds to the median probability. [file 12859_2022_4608_MOESM1_ESM.pdf]

Additional File 1

Table S1: Effects of Downsampling per Entity Type in Hetionet.

Type	Original	(%)	Modified	(%)	Change	(%)
Anatomy	400	0.88	394	2.03	6	+129.70
Biological Pr.	11381	25.20	1668	8.61	9713	-65.82
Cellular C.	1391	3.08	0	0.00	1391	-100.
**Compound**	1538	3.40	1503	7.76	35	**+127.89**
**Disease**	136	0.30	136	0.70	0	**+133.20**
Gene	19145	42.39	11291	58.30	7854	+37.53
Molecular F.	2884	6.38	932	4.81	1952	-24.63
Pathway	1822	4.03	959	4.95	863	+22.74
Pharmacologic C.	345	0.76	252	1.30	93	+70.34
Side Effect	5701	12.62	1833	9.46	3868	-25.01
Symptom	415	0.91	396	2.04	19	+122.52
Total	45158	100	19364	100		-

Table S2: Effects of Downsampling per Entity Type in DRKG.

Type	Original	(%)	Modified	(%)	Change	(%)
Anatomy	400	0.41	391	0.54	9	+31.47
Atc	4048	4.21	3262	4.56	786	+8.38
Biological Pr.	11381	11.80	6551	9.16	4830	-22.58
Cellular C.	1391	1.44	0	0.00	1391	-100
**Compound**	23347	24.28	19633	27.47	3714	**+13.10**
**Disease**	4952	5.15	4813	6.73	139	**+30.72**
Gene	39220	40.80	27380	38.31	11840	-6.10
Molecular F.	2884	3.00	2338	3.2	546	+9.034
Pathway	1822	1.89	1745	2.44	77	+28.81
Pharmacologic C.	345	0.35	344	0.48	1	+34.10
Side Effect	5701	5.93	4619	6.46	1082	+8.97
Symptom	415	0.43	391	0.54	24	+26.71
Tax	215	0.22	0	0.00	215	-100
Total	96121	100	71467	100		-

Table S3: Effects of Downsampling on the ranking of antiviral compounds against SARS CoV2-entities.

	Favipiravir			Danoprevir			Lopinavir			Ritonavir		
Disease entity	Original	Modified	Change	Original	Modified	Change	Original	Modified	Change	Original	Modified	Change
SARS-CoV2E	8860	3904	-4956	3016	3649	+633	6641	1491	-5150	1562	1304	-258
SARS-CoV2M	4220	8475	4255	5689	3363	-2325	284	1363	+1079	2568	1359	-1209
SARS-CoV2N	5265	4198	-1067	5141	2385	-2756	482	3183	+2700	657	509	-147
**SARS-CoV2Spike**	**13145**	**4766**	**-8378**	**6799**	**3750**	**-3049**	**1835**	**1828**	**-7**	**578**	**1574**	**+996**
SARS-CoV2nsp1	4387	4961	573	3926	2365	-1561	2474	1262	-1212	2108	398	-1710
SARS-CoV2nsp10	3771	5260	+1488	1259	2365	+1105	418	697	+279	316	806	+490
SARS-CoV2nsp11	5388	6192	+803	3531	2548	-983	4783	386	-4397	2100	969	-1131
SARS-CoV2nsp12	10169	7406	-2763	19394	2738	-16656	1409	549	-859	479	1948	1469
SARS-CoV2nsp13	3760	4077	+317	5925	4482	-1442	808	2883	+2075	693	2286	+1593
SARS-CoV2nsp14	12839	4893	-7945	4609	1684	-2925	2875	788	-2087	72	1217	+1145
SARS-CoV2nsp15	6942	4363	-2579	2491	1393	-1098	685	485	-199	166	189	+23
SARS-CoV2nsp2	9731	4454	-5277	11542	2139	-9403	259	1005	+745	1889	336	-1553
SARS-CoV2nsp4	10764	6022	-4742	2512	4323	+1811	2936	2747	-189	2783	753	-2030
SARS-CoV2nsp5	11219	4039	-7179	2659	2892	+232	1246	305	-941	317	2052	+1735
SARS-CoV2nsp5 C145A	5458	5906	+448	4392	3279	-1113	1987	174	-1813	239	457	+217
SARS-CoV2nsp6	3022	5108	+2085	3398	3755	+357	3382	560	-2822	477	1176	+699
SARS-CoV2nsp7	6236	4405	-1831	14625	3444	-11181	6579	80	-6499	254	865	+611
SARS-CoV2nsp8	5237	4266	-971	3755	2580	-1175	3552	1039	-2513	2053	200	-1852
SARS-CoV2nsp9	8079	4978	-3100	5432	2171	-3260	2561	1561	-1000	2718	199	-2519
SARS-CoV2orf10	6404	4524	-1879	1200	3733	+2533	735	1791	+1056	1776	173	-1603
SARS-CoV2orf3a	3512	5467	+1955	1798	2742	+944	1829	540	-1289	740	2291	+1550
SARS-CoV2orf3b	2867	5319	+2452	2558	3159	+600	642	342	-299	608	850	+242
SARS-CoV2orf6	3823	4710	+886	10305	2257	-8048	642	652	+10	3118	726	-2392
SARS-CoV2orf7a	9725	5961	-3763	1425	2820	+1394	3573	2072	-1500	781	695	-85
SARS-CoV2orf8	4302	6508	+2206	3032	1533	-1499	286	2669	+2383	1375	4430	+3055
SARS-CoV2orf9b	8684	6677	-2006	2747	2012	-734	116	1791	+1674	1028	1319	+291
SARS-CoV2orf9c	4566	5980	+1414	7251	4944	-2307	10332	2753	-7578	1318	2386	+1068
**Mean**	**6754**	**5289**	**-1465**	**5200**	**2907**	**-2292**	**2346**	**1296**	**-1050**	**1213**	**1165**	**-48**

Shown is the rank of a given SARS-CoV2 entity with a given antiviral compound when the model is trained on the full dataset versus when it is trained on the filtered dataset. The SARS-COV2 entities are ranked against all remaining 19,633 compound entities by connecting them with a "treats" relation. Rank 1 means the highest probability that the two entities are connected by a "treats" relation, rank 19,633 means the lowest probability, rank 9,816 corresponds to the median probability.
