# Supplementary material for: Task-driven knowledge graph filtering improves prioritizing drugs for repurposing
Source: BMC Bioinformatics. 2022 Mar 4;23:84. doi: 10.1186/s12859-022-04608-y (PMC8894843; doi:10.1186/s12859-022-04608-y)
Supplement: Supplementary file 2 — Additional file 2: Table S4. Metapath Selection. Metapaths that have been selected for random metapath walking. Denoted are the entity types traversed. C: Compound, D: Disease, G: Gene, A: Anatomy, PC: Pharmacologic Class, S: Symptom, BP: Biological Process, MF: Metabolic Function, PW: Pathway, SE: Side Effect. Table S5: Additional Metapaths for DRKG. Metapaths selected for DRKG in addition to the metapaths denoted in Table S4. C: Compound, Atc: Atc, D: Disease, G: Gene. [file 12859_2022_4608_MOESM2_ESM.pdf]

Additional File 2

Table S4: Metapath Selection

C - C - C - C - D	C - C - C - D
C - C - C - G - D	C - C - D
C - C - D - C - D	C - C - G - A - D
C - C - G - C - D	C - C - G - D
C - C - G - D - D	C - C - G - G - D
C - C - PC - C - D	C - D - C - D
C - D - C - D - D	C - D - D
C - D - D - A - D	C - D - D - S - D
C - G - A - D	C - G - A - D - D
C - G - A - G - D	C - G - BP - G - D
C - G - C - C - D	C - G - C - D
C - G - C - D - D	C - G - C - G - D
C - G - D	C - G - D - A - D
C - G - D - D	C - G - D - D - D
C - G - D - G - D	C - G - D - S - D
C - G - G - A - D	C - G - G - C - D
C - G - G - D	C - G - G - D - D
C - G - G - G - D	C - G - MF - G - D
C - G - PW - G - D	C - PC - C - C - D
C - PC - C - D	C - PC - C - G - D
C - SE - C - D	C - SE - C - D - D
C - SE - C - G - D	

Metapaths that have been selected for random metapath walking. Denoted are the entity types traversed.
C: Compound, D: Disease, G: Gene, A: Anatomy, PC: Pharmacologic Class, S: Symptom,
BP: Biological Process, MF: Metabolic Function, PW: Pathway, SE: Side Effect

Table S5: Additional Metapaths for DRKG

C - Atc - C - D	C - Atc - C - D - D
C - Atc - C - G - D	

Metapaths selected for DRKG in addition to the metapaths denoted in Tab. S4.
C: Compound, Atc: Atc, D: Disease, G: Gene
