# Supplementary material for: Task-driven knowledge graph filtering improves prioritizing drugs for repurposing
Source: BMC Bioinformatics. 2022 Mar 4;23:84. doi: 10.1186/s12859-022-04608-y (PMC8894843; doi:10.1186/s12859-022-04608-y)
Supplement: Supplementary file 3 — Additional file 3: Table S6. Additional performance metrics for Hetionet. Table S7: Additional performance metrics for DRKG. Table S8: Compound and Disease entities that are disconnected by an Ablation Experiment. Table S9: Entity Type Ablation Experiments—Hetionet—TransE. Table S10: Entity Type Ablation Experiments—Hetionet—ComplEx. Table S11: Entity Type Ablation Experiments—DRKG—TransE. Table S12: Entity Type Ablation Experiments—DRKG—ComplEx. Table S13: Effects of train/valid/test split on model performance—TransE. Table S14: Effects of train/valid/test split on model performance—ComplEx. Table S15: Additional performance metrics for the Cancer Case Study. Table S16: Sizes of training, validation and test splits. [file 12859_2022_4608_MOESM3_ESM.pdf]

Additional File 3

Additional Performance Metrics

In addition to Mean Reciprocal Rank (MRR) which is described and displayed in the main manuscript, several other metrics have been measured to gain a more complete picture of model performance (see Tables S6 & S7). [1]

Mean Reciprocal Rank

$$MRR = \frac{1}{|Q|}\sum_{i=1}^{Q}\frac{1}{rank_i} \tag{1}$$

MRR captures performance over the whole range of possible ranks but is biased towards top-end performance, meaning low-ranking true predictions. It lies on the interval $[0, 1]$ where closer to 1 (higher) is better.

Mean Rank

$$MeanRank = \frac{1}{|Q|}\sum_{i=1}^{Q} rank_i \tag{2}$$

The mean rank is easy to interpret and captures performance over the whole range of possible ranks. However, the judgment if a given mean rank is good or bad depends on the number of possible total ranks. It lies on the interval $[1, \infty]$ where closer to 1 (lower) is better.

Hits@K

$$Hits@K = \frac{1}{|Q|}\sum_{i=1}^{Q}[rank_i < k] \tag{3}$$

Hits@K measures how many of the true results get ranked into the top k results. This metric is useful to evaluate top-end performance but does not capture if a true example fails to get ranked into the top k by a little or by a large margin. It lies on the interval $[0, 1]$ where closer to 1 (higher) is better.

Entity Type Ablation Experiments

To see how the removal of all entities of a single type influences the performance of the model, each entity type that is adjacent to compounds and diseases has been omitted in one experiment each. Each experiment consists of an individual HPO, reported is the performance of the best performing HPO trial on the same test data that was also used for the other ablation experiments. When entities and their incident relations are removed from the graph, their adjacent entities also lose an incident relation. Some compounds and diseases which previously have been connected only to an entity that has been removed in an ablation experiment are then disconnected, i.e. without connection of the rest of the graph. They are still used for the evaluation of drug repurposing performance, however, since they are not incident to a $treats$ relation in the training set by definition, they all are negative samples during training. The percentage of compound and disease entities which are part of the evaluation but are disconnected by an ablation experiment is shown in Table S8. The link prediction performance results of the ablation experiments are compared to the full dataset (Original) and the dataset that has been modified with our metapath filtering approach (Modified) (see Tables S9 – 12).

Split Effect Experiments

In all our experiments, relations of type "treats" have been randomly assigned to a train, test and validation set. To assess the effect that this assignemt has on the model performance, we have applied 4 different splits of Hetionet, where in each split, a different 25% of the relations has been held out, half of which have been used for hyperparameter search and half of which have been used for the assessment of the final test performance. For each split, after setting the holdout set aside, an independent modified version of the graph has been produced by applying our metapath-based filtering approach on each of them. Then, for each of the four original and modified graphs, an exhaustive HPO has been carried out. Reported is the test set performance of the best performing HPO trial per split (see Tables S13 & S14).

[1] For more information about KGE performance evaluation see
https://pykeen.readthedocs.io/en/stable/tutorial/understanding_evaluation.html#rank-based-metrics

Table S6: Additional performance metrics of the best performing models on Hetionet

Model	TransE			Distmult		
Metric	Original	Modified	Change	Original	Modified	Change
MRR	0.223	0.237	+6.4%	0.228	0.251	+9.3%
Mean Rank	98	95	-4	97	96	-1
Hits@5	0.287	0.32	+14.8%	0.335	0.311	+4.5%
Hits@10	0.377	0.457	+21.1%	0.404	0.425	+5.0%
Hits@20	0.526	0.574	+9.1%	0.500	0.553	+9.6%
Hits@50	0.707	0.712	+0.7%	0.670	0.696	+3.8%
Model	ComplEx			RESCAL		
Metric	Original	Modified	Change	Original	Modified	Change
MRR	0.197	0.278	+40.8%	0.242	0.294	+21.1%
Mean Rank	95	82	-13	74	55	-19
Hits@5	0.292	0.356	+21.8%	0.340	0.372	+9.3%
Hits@10	0.382	0.452	+18.0%	0.441	0.510	+15.6%
Hits@20	0.500	0.579	+15.9%	0.521	0.611	+17.3%
Hits@50	0.676	0.702	+3.9%	0.680	0.803	+17.9%
Model	ConvE					
Metric	Original	Modified	Change			
MRR	0.131	0.165	+25.9%			
Mean Rank	179	136	-43			
Hits@5	0.175	0.207	+18.1%			
Hits@10	0.255	0.276	+8.3%			
Hits@20	0.340	0.420	+23.4%			
Hits@50	0.521	0.601	+15.3%			

Table S7: Additional performance metrics of the best performing models on DRKG

Model	TransE			Distmult		
Metric	Original	Modified	Change	Original	Modified	Change
MRR	0.083	0.093	+12.0%	0.089	0.093	+3.6%
Mean Rank	580	536	-44	625	620	-5
Hits@5	0.114	0.124	+8.7%	0.127	0.130	+2.2%
Hits@10	0.170	0.180	+5.8%	0.186	0.186	+0.0%
Hits@20	0.245	0.252	+2.8%	0.262	0.259	-1.3%
Hits@50	0.370	0.380	+2.7%	0.386	0.383	-0.9%
Model	ComplEx			RESCAL		
Metric	Original	Modified	Change	Original	Modified	Change
MRR	0.089	0.095	+5.9%	0.057	0.65	+12.4%
Mean Rank	609	606	-3	843	834	-9
Hits@5	0.122	0.128	+4.8%	0.076	0.084	+10.5%
Hits@10	0.180	0.190	+5.1%	0.091	0.096	+5.4%
Hits@20	0.255	0.260	+2.1%	0.152	0.157	+3.2%
Hits@50	0.377	0.387	+2.6%	0.268	0.271	+1.1%
Model	ConvE					
Metric	Original	Modified	Change			
MRR	0.061	0.065	+4.9%			
Mean Rank	845	840	-5			
Hits@5	0.083	0.085	+2.4%			
Hits@10	0.096	0.099	+3.1%			
Hits@20	0.183	0.185	+1.0%			
Hits@50	0.276	0.280	+1.4%			

Table S8: Compound and Disease entities that are disconnected by an Ablation Experiment

Dataset	Type	Original	No Anat.	No Gene	No P.C.	No S.E.	No Sympt.	Modified
Hetionet	Compound	0%	0.	2.9%	0%	0.2%	0%	0%
	Disease	0%	0%	1.4%	0%	0%	0%	0%
DRKG	Compound	0%	0%	52.0%	0%	0%	0%	0%
	Disease	0%	0%	25.1%	0%	0%	0%	0%

No Anat.: No Anatomy, No P.C.: No Pharmacologic Class, No S.E.: No Side Effect, No Sympt: No Symptom.

Table S9: Entity Type Ablation Experiments - Hetionet - TransE

Metric	Original	No Anat.	No Gene	No P.C.	No S.E.	No Sympt.	Modified
MRR	0.223	0.214	0.338	0.222	0.181	0.198	0.237
Mean Rank	98	97	75	105	141	99	95
Hits@5	0.287	0.287	0.436	0.313	0.25	0.260	0.32
Hits@10	0.377	0.382	0.526	0.382	0.351	0.340	0.457
Hits@20	0.526	0.5	0.622	0.478	0.473	0.457	0.574
Hits@50	0.707	0.64	0.77	0.622	0.617	0.643	0.712

No Anat.: No Anatomy, No P.C.: No Pharmacologic Class, No S.E.: No Side Effect, No Sympt: No Symptom.

Table S10: Entity Type Ablation Experiments - Hetionet - ComplEx

Metric	Original	No Anat.	No Gene	No P.C.	No S.E.	No Sympt.	Modified
MRR	0.197	0.218	0.355	0.284	0.141	0.229	0.278
Mean Rank	95	103	49	91	156	100	82
Hits@5	0.292	0.329	0.473	0.404	0.202	0.303	0.356
Hits@10	0.382	0.404	0.563	0.478	0.281	0.398	0.452
Hits@20	0.500	0.521	0.696	0.563	0.393	0.510	0.579
Hits@50	0.676	0.670	0.840	0.696	0.558	0.633	0.702

No Anat.: No Anatomy, No P.C.: No Pharmacologic Class, No S.E.: No Side Effect, No Sympt: No Symptom.

Table S11: Entity Type Ablation Experiments - DRKG - TransE

Metric	Original	No Anat.	No Gene	No P.C.	No S.E.	No Sympt.	Modified
MRR	0.083	0.084	0.095	0.082	0.086	0.089	0.093
Mean Rank	580	566	3123	560	556	544	536
Hits@5	0.114	0.113	0.128	0.110	0.115	0.121	0.124
Hits@10	0.170	0.162	0.179	0.165	0.169	0.175	0.180
Hits@20	0.245	0.234	0.251	0.232	0.242	0.246	0.252
Hits@50	0.370	0.358	0.377	0.359	0.364	0.374	0.380

No Anat.: No Anatomy, No P.C.: No Pharmacologic Class, No S.E.: No Side Effect, No Sympt: No Symptom.

Table S12: Entity Type Ablation Experiments - DRKG - ComplEx

Metric	Original	No Anat.	No Gene	No P.C.	No S.E.	No Sympt.	Modified
MRR	0.089	0.088	0.097	0.091	0.088	0.085	0.095
Mean Rank	609	607	1294	611	618	639	606
Hits@5	0.122	0.122	0.131	0.126	0.121	0.116	0.128
Hits@10	0.180	0.182	0.190	0.185	0.177	0.172	0.190
Hits@20	0.255	0.256	0.261	0.259	0.252	0.248	0.260
Hits@50	0.377	0.381	0.381	0.386	0.381	0.367	0.387

No Anat.: No Anatomy, No P.C.: No Pharmacologic Class, No S.E.: No Side Effect, No Sympt: No Symptom.

Table S13: Effects of train/valid/test split on model performance - TransE

	Split 1			Split 2		
Metric	Original	Modified	Change	Original	Modified	Change
MRR	0.223	0.237	+6.4%	0.257	0.31	+21.7%
Mean Rank	98	95	-4	87	69	-18
Hits@5	0.287	0.32	+14.8%	0.336	0.394	+17.2%
Hits@10	0.377	0.457	+21.1%	0.410	0.463	+12.8%
Hits@20	0.526	0.574	+9.1%	0.552	0.578	+4.7%
Hits@50	0.707	0.712	+0.7%	0.710	0.747	+5.1%
	**Split 3**			**Split 4**		
Metric	Original	Modified	Change	Original	Modified	Change
MRR	0.220	0.24	+11.1%	0.188	0.22	+18.4%
Mean Rank	103	79	-24	108	101	-7
Hits@5	0.321	0.326	+1.6%	0.284	0.3368	+18.5%
Hits@10	0.394	0.447	+13.3%	0.342	0.405	+18.4%
Hits@20	0.494	0.573	+15.9%	0.473	0.531	+12.2%
Hits@50	0.642	0.715	+11.4%	0.663	0.7	+5.55%

Table S14: Effects of train/valid/test split on model performance - Complex

	Split 1			Split 2		
Metric	Original	Modified	Change	Original	Modified	Change
MRR	0.197	0.278	+40.8%	0.249	0.287	+15.2%
Mean Rank	95	82	-13	87	67	-20
Hits@5	0.292	0.356	+21.8%	0.315	0.415	+31.6%
Hits@10	0.382	0.452	+18%	0.426	0.494	+16.0%
Hits@20	0.500	0.579	+15.9%	0.547	0.631	+15.3%
Hits@50	0.676	0.702	+3.9%	0.710	0.789	+11.1%
	Split 3			Split 4		
Metric	Original	Modified	Change	Original	Modified	Change
MRR	0.258	0.274	+6.1%	0.217	0.214	-1.3%
Mean Rank	94	70	-24	86	75	-11
Hits@5	0.368	0.257	-2.8%	0.294	0.300	+1.7%
Hits@10	0.463	0.457	-1.1%	0.415	0.421	+1.2%
Hits@20	0.557	0.584	+4.7%	0.536	0.542	+1.0%
Hits@50	0.700	0.721	+3.0%	0.705	0.673	-4.4%

Table S15: Additional performance metrics for the Cancer Case Study

Disease	Cancer (general)			Breast Cancer		
Metric	Original	Modified	Change	Original	Modified	Change
MRR	0.0154	0.0189	+22.7%	0.0137	0.0245	+78.8%
Mean Rank	2188	2382	+194	1483	1821	+338
Hits@5	0.0070	0.0141	+101.4%	0.0	0.0153	$+\infty$%
Hits@10	0.0283	0.0496	+75.2%	0.0461	0.0923	+102.2%
Hits@20	0.0496	0.0709	+42.9%	0.1076	0.1230	+14.3%
Hits@50	0.1063	0.1276	+20.0%	0.1384	0.1538	+11.1%
Disease	Lung Cancer			Colon Cancer		
Metric	Original	Modified	Change	Original	Modified	Change
MRR	0.0177	0.0355	+100.5%	0.0144	0.0160	+14.2%
Mean Rank	1354	1237	-117	1776	1886	+110
Hits@5	0.0	0.0588	$+\infty$%	0.0	0.0625	$+\infty$%
Hits@10	0.0588	0.0588	+0.0%	0.0625	0.0625	+0.0%
Hits@20	0.1176	0.0588	-50.0%	0.0625	0.0625	+0.0%
Hits@50	0.1176	0.1764	+50.%	0.125	0.0625	-50%

Table S16: Sizes of training, validation and test splits. All figures are before introduction of inverse relations.

Dataset Version	Training	o/w Treats	Validation	o/w Treats	Test	o/w Treats
Hetionet Fold1 Orig.	2,249,619	567	94	94	94	94
Hetionet Fold1 Mod.	1,516,611	567	94	94	94	94
Hetionet Fold2 Orig.	2,249,618	566	94	94	95	95
Hetionet Fold2 Mod.	1,516,960	566	94	94	95	95
Hetionet Fold3 Orig.	2,249,618	566	94	94	95	95
Hetionet Fold3 Mod.	1,510,654	566	94	94	95	95
Hetionet Fold4 Orig.	2,249,618	566	94	94	95	95
Hetionet Fold4 Mod.	1,510,981	566	94	94	95	95
DRKG Orig.	5,838,613	48,247	5,939	5,939	5,951	5,951
DRKG Mod.	5,473,200	48,174	5,939	5,939	5,951	5,951
